# Relmacabtagene autoleucel (relma‐cel) CD19 CAR‐T therapy for adults with heavily pretreated relapsed/refractory large B‐cell lymphoma in China

**DOI:** 10.1002/cam4.3686

**Published:** 2020-12-31

**Authors:** Zhitao Ying, Haiyan Yang, Ye Guo, Wenyu Li, Dehui Zou, Daobin Zhou, Zhao Wang, Mingzhi Zhang, Jianqiu Wu, Hui Liu, Pian Zhang, Su Yang, Zisong Zhou, Hongxia Zheng, Yuqin Song, Jun Zhu

**Affiliations:** ^1^ Department of Lymphoma Key Laboratory of Carcinogenesis and Translational Research (Ministry of Education/Beijing) Peking University Cancer Hospital & Institute Beijing China; ^2^ Department of Lymphatic Medical Cancer Hospital of the University of Chinese Academy of Sciences Zhejiang China; ^3^ Department of Oncology Shanghai East Hospital Shanghai China; ^4^ Department of Lymphoma Guangdong Academy of Medical Sciences Guangdong Provincial People’s Hospital Guangdong China; ^5^ Lymphoma Center Institute of Hematology & Blood Diseases Hospital Chinese Academy of Medical Sciences & Peking Union Medical College Institute of Hematology and Blood Diseases Hospital (IH) Tianjin China; ^6^ Department of Hematology Peking Union Medical College Hospital Chinese Academy of Medical Sciences Beijing China; ^7^ Department of Hematology Beijing Friendship Hospital Capital Medical University Beijing China; ^8^ Department of Oncology the First Affiliated Hospital of Zhengzhou University Zhengzhou China; ^9^ Department of Medical Oncology Jiangsu Institute of Cancer Research Jiangsu Red Cross Cancer Center Jiangsu Cancer Hospital the Affiliated Hospital of Nanjing Medical University Nanjing China; ^10^ Department of Hematology Beijing Hospital Beijing China; ^11^ JW Therapeutics (Shanghai) Co. Ltd Shanghai China

**Keywords:** CAR‐T, CD19, cellular kinetics, LBCL, Relma‐cel

## Abstract

**Background:**

Despite numerous chimeric antigen receptor T‐cell (CAR‐T) trials conducted in China, no CAR‐T has been registered in the country. Furthermore, China law and regulations restrict the export of patient material for CAR‐T manufacture abroad. Relma‐cel (JWCAR029), an anti‐CD19 product produced with a commercial‐ready process in China, was evaluated in the first prospective, single‐arm, multicenter, pivotal study of CAR‐T therapy conducted under Chinese IND to support an NMPA‐accepted BLA submission in relapsed/refractory (r/r) LBCL (NCT04089215).

**Methods:**

Patients were randomized to receive either 100 × 10^6^ (low dose, *n* = 27) or 150 × 10^6^ (high dose, *n* = 32) CAR+ T‐cells as a single infusion following lymphodepleting chemotherapy (fludarabine 25 mg/m^2^ and cyclophosphamide 250 mg/m^2^ daily × 3), and then, monitored for efficacy and safety outcomes and pharmacokinetics. The primary endpoint was ORR at 3 months, as assessed by the investigators. Secondary endpoints included DOR, PFS, OS, and adverse event frequency/severity and cell expansion kinetics.

**Results:**

As of the data cutoff on 17 June 2020, 68 patients were enrolled, and 59 were treated. Among the 58 efficacy‐evaluable patients, the primary endpoint of 3 month ORR was 60.3% (95% CI, 46.6–73.0), excluding the null hypothesis rate of 20%. Any grade and severe grade CRS occurred in 47.5% and 5.1%, respectively, and any grade and severe grade neurotoxicity events occurred in 20.3% and 5.1%.

**Conclusions:**

Relma‐cel met the primary endpoint analysis and demonstrated a high rate of durable responses and low rate of CAR‐T‐associated toxicities in patients with r/r LBCL in a multicenter trial supporting regulatory submission in China.

## Graphical abstract

The RELIANCE Trial provided the first demonstration of licensure‐quality CAR‐T manufacturing and clinical trial data generation in r/r patients originating in China. The results with relma‐cel demonstrate similar preliminary response rates and PK profiles while providing the potential for an improved toxicity profile in heavily‐pre‐treated patients with r/r DLBCL having poor risk features relative to other CD19‐specific CAR‐Ts approved in the US and EU.

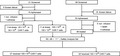

## 1 INTRODUCTION

Non‐Hodgkin's lymphoma (NHL) was diagnosed in over a half million persons worldwide in 2018.^1^ In China, 68,500 new cases, 37,600 deaths and 237,000 existing cases of NHL were estimated in 2016.^2^ Chinese large B‐cell lymphoma (LBCL) patients typically receive R‐CHOP as frontline therapy. For those with relapsed or refractory disease, salvage chemotherapy regimens followed by high‐dose chemotherapy and autologous stem‐cell transplant (ASCT) is a common strategy, however, only half of those with chemo‐responsive disease receive ASCT in China.^3^, ^4^ Once failing second‐line therapy, these patients have few therapeutic options beyond additional cycles of salvage chemotherapy, which has generally been associated with low response rates and limited overall survival.^5^, ^6^

Commercially available CAR‐T cell products targeting CD19 have been available in the United States (US) since 2017 and in European Union (EU) since 2018 for patients with r/r DLBCL after failing two prior lines of therapy. These products, including axicabtagene ciloleucel and tisagenlecleucel, have demonstrated high rates of durable disease response in registrational clinical trials.^7^, ^8^ However, neither of these products, nor any other CAR‐T product has been approved for commercial use for r/r LBCL in China, despite the country having the second largest number of CAR‐T trials after the US. This is due to two primary factors: (a) Most CAR‐T trials in China are single‐center studies not conducted under a Clinical Trials Application (CTA) with National Medical Products Administration (NMPA), and thus, the data are not able to support product registration, and (b) unlike the EU, national laws and regulations prohibit the export of patient material, such as an apheresed peripheral blood mononuclear cells, that would enable access to commercial CAR‐T manufacturing outside the country to provide early patient earlier access to these agents.

Relma‐cel is a CD19‐targeted, second generation CAR‐T cell product with a 4‐1BB costimulatory domain manufactured in China. In fact, relma‐cel expresses the same CAR as another CAR‐T manufactured in the US, lisocabtagene maraleucel, which has demonstrated high response rates in r/r LBCL with low levels of CAR‐T‐associated toxicity.^9^, ^10^, ^11^ However, relma‐cel uses a commercial‐ready process developed in China that does not require separate CD4 and CD8 T‐cell production trains to provide a wide range of doses with consistent product attributes.^12^

A phase I study of relma‐cel (NCT03344367 and NCT03355859) demonstrated preliminary safety and efficacy in r/r NHL patients over a dosing range from 25 million to 150 million CAR+ T‐cells.^13^, ^14^, ^15^ These data supported the design and execution of the first prospective, single‐arm, multicenter, registrational study of CAR‐T therapy conducted under Chinese Investigational New Drug (IND) to enable an NMPA‐accepted Biologic License Application (BLA) submission in r/r LBCL (NCT04089215). Herein, the results of this pivotal trial in China are reported.

## 2 MATERIALS AND METHODS

### 2.1 Study design

**FIGURE 1:**
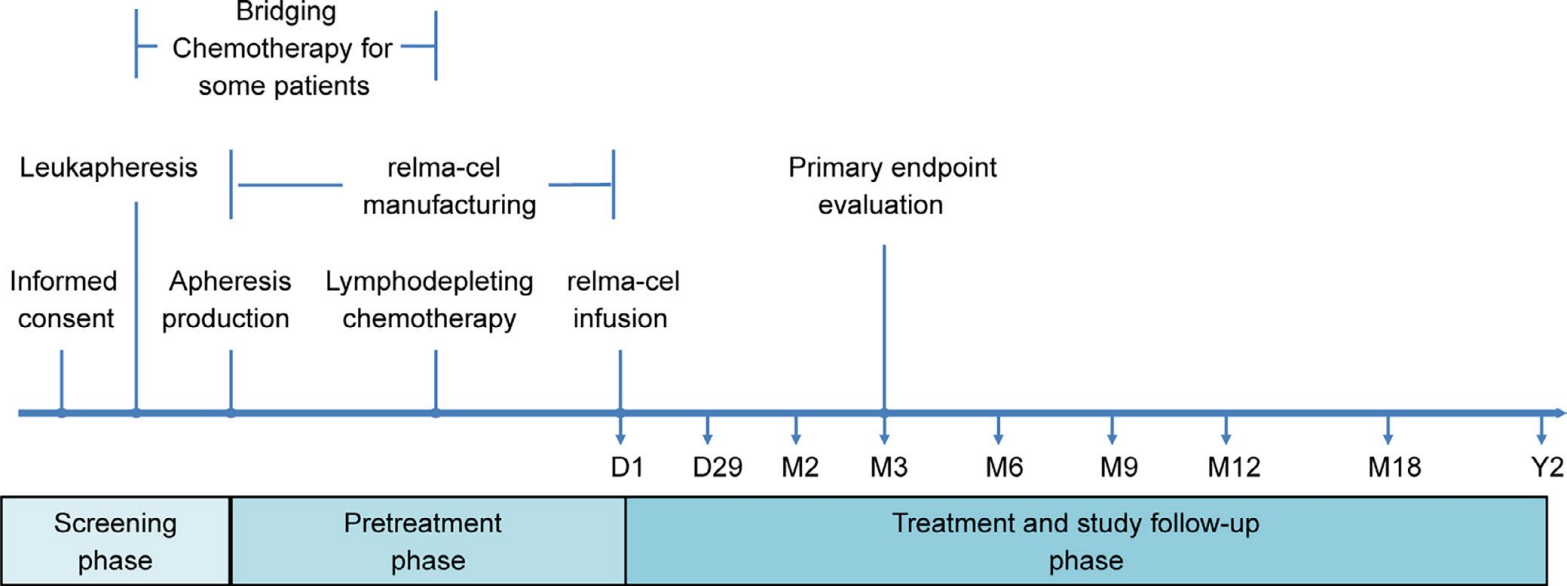
The study schema for RELIANCE Trial demonstrates the sequential phases for enrolled patients including screening phase, pretreatment phase, and treatment and study follow‐up phase. D, day; M, month; Y, year.

This was a single‐arm, open‐label, multicenter clinical trial design evaluating relma‐cel in adult (≥18 years) patients with CD19+ r/r LBCL, study schema provided in Figure 1. This study was approved by Ethics Committee at each site and conducted in accordance with Good Clinical Practice guidelines of the International Committee on Harmonization. After providing informed consent, patients were randomized to receive either 100 × 10^6^ (low dose, *n* = 27) or 150 × 10^6^ (high dose, *n* = 32) CAR+ T‐cells. Leukapheresed patients could receive bridging chemotherapy based on investigator assessment of disease burden and tumor progression risk. Lymphodepleting (LD) chemotherapy was administered over 3 days, including fludarabine (25 mg/m^2^ i.v. daily) and cyclophosphamide (250 mg/m^2^ i.v. daily), followed 2–7 days later by a single intravenous infusion of relma‐cel at the assigned dose. Efficacy was assessed at baseline, 1, 3, 9, 12, 18, and 24 months post infusion. Individuals receiving bridging chemotherapy were restaged prior to beginning LD chemotherapy. Safety events were monitored from beginning of LD through 2 years follow‐up.

### 2.2 Participant population

Eligible adult patients had measurable, histologically confirmed r/r LBCL, including several LBCL subtypes (nonspecific DLBCL [DLBCL‐NOS], high‐grade B‐cell lymphoma with *MYC* and *BCL2*, and/or *BCL6* rearrangements [double‐/triple‐hit lymphoma, DHL/THL], primary mediastinal Large B‐cell lymphoma [PMBCL], grade 3b follicular lymphoma [FL], and FL‐transformed DLBCL) on the basis of the 2008 WHO Classification,^16^ and not had allogeneic transplant within 90 days, or primary central nervous system (CNS) lymphoma. Refractory or relapsed patients must have previously been treated with at least two lines of therapy containing an anti‐CD20 monoclonal antibody and anthracycline‐containing regimen. Those receiving ASCT must have relapsed, progressed disease or never achieved remission within 24 months posttransplant.

### 2.3 CAR construct and CAR‐T cell manufacturing

Relma‐cel is comprised of autologous CD4+ and CD8+ T‐cells that have been transduced to express a CD19‐specific chimeric antigen receptor (CAR) and a truncated epidermal growth factor receptor (EGFRt). The CAR receptor is comprised of an extracellular scFv‐binding domain derived from a murine CD19‐specific hybridoma cell line (FMC63), fused to the 4‐1BB and CD3 ζ chain signaling domains. Autologous mononuclear cells (MNCs) are collected from patient via leukapheresis. The T‐cells are specifically enriched from apheresis using CD4 and CD8 microbeads. The selected T‐cells are activated using CD3/CD28 microbeads. The activated T‐cells are transduced ex vivo with nonreplicative self‐inactivating (SIN) lentiviral vector containing the CAR transgene. The transduced T‐cells are expanded in cell culture. The expanded CAR‐T cells are harvested and formulated into a cryopreservation medium. The drug product consists of a single frozen cell suspension formulated in a cryopreservation medium.

### 2.4 Outcomes

The primary endpoint was the proportion of patients achieving objective response at 3 month by investigator assessment, using the Lugano Classification.^17^ Secondary endpoints were best objective response (BOR), complete response rate (CRR), duration of response (DOR), progression free survival (PFS), and overall survival (OS; Lugano criteria, 2014), incidence of adverse events (Cytokine Release Syndrome [CRS] by Lee 2014, and all other events by Common Terminology Criteria for Adverse Events [CTCAE] v4.03). Exploratory endpoints included concentrations of eight serum cytokines (interleukin‐2 [IL‐2], IL‐6, IL‐8, IL‐15, transforming growth factor‐β1 [TGF‐β1], tumor necrosis factor alpha [TNF‐α], interferon gamma [IFN‐γ], and monocyte chemotactic protein 1[MCP‐1]) and two serum biomarkers (C‐reactive protein [CRP] and ferritin; by Meso Scale Discovery [MSD] and Enzyme‐linked Immunosorbent Assay [ELISA]), serum anti‐therapeutic antibodies (ATA; by MSD), and CAR‐T cell concentrations in the peripheral blood (PB) using transgene copy number per microgram DNA (by qPCR) and CD3+ CAR+ cell frequency (by flow cytometry). ATA testing uses antibody targeting the scFV part of CAR Extracellular domain.

### 2.5 Statistical analysis

The primary analysis was conducted based on the data cutoff on 17 June 2020 with 58 patients assessed for 3 month objective response rate (ORR) in the efficacy analysis set. Under the null hypothesis assumption of ORR ≤20% for standard therapy in this setting and an alternative hypothesis assumption of 40% ORR, with the sample size of 58 patients, this study has ≥92% power and one‐sided *α* of 0.025.

DOR, PFS, and OS were summarized using Kaplan–Meier methods.^18^ DOR was defined as time of first response to progression or death. PFS was defined as the time from infusion to disease progression or death and OS was defined as the time from the infusion to death. Patients were censored either at the relma‐cel infusion date, if no radiologic assessment post infusion, or the date of last tumor assessment prior to starting new treatment before PD, if follow‐up monitoring was discontinued beyond PD or death, or if death or PD occurred after ≥2 consecutive missed restaging visits.

Pharmacokinetics (PK) parameters were calculated using non‐compartmental analyses with Phoenix WinNonlin 8.0 (Certara) for Cmax (observed maximum plasma concentration following infusion), Tmax (time at which maximum plasma concentration was observed), and AUC_1‐29_ (exposure calculated as the product of plasma concentration and time using a trapezoid rule). Boxplots of PK parameters analyzed by age, sex, ATA positive or negative post infusion, and tocilizumab or steroid use; nonresponse, OR or CR; and CRS or neurotoxicity (NT) by severity grade were calculated using Wilcoxon test.

## 3 RESULTS

### 3.1 Patient characteristics

**FIGURE 2:**
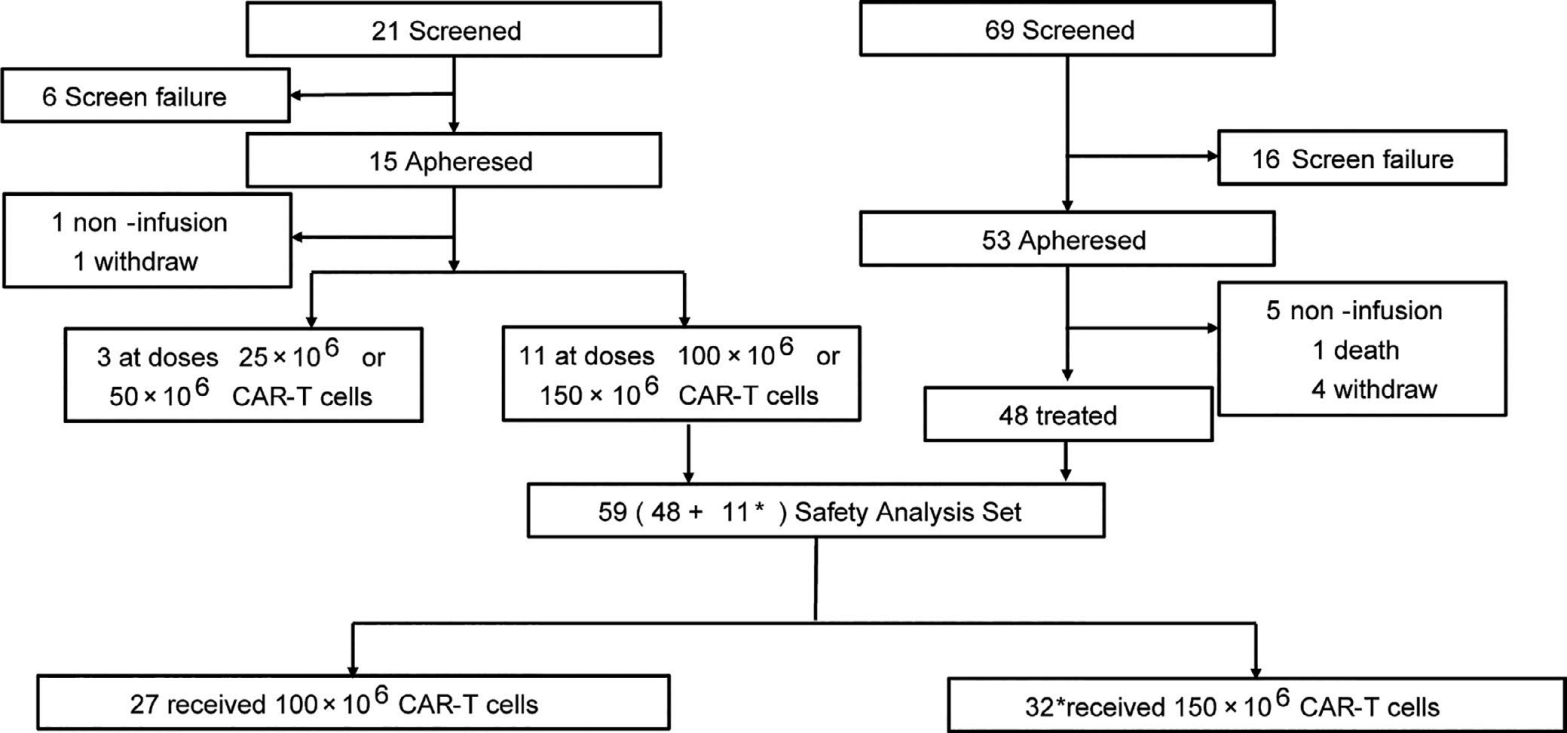
Patients screening, enrollment and treatment. Ninety patients were screened, 68 apheresed, and 59 treated. Among the six patients who were not received relma‐cel, one died before infusion, five withdraw. *One patient, excluded from efficacy analysis set, received relma‐cel infusion that did not meet potency threshold criterion, but achieved CR by D29 that continued for more than 1 year.

Between Nov, 2017 and Dec, 2019, 90 patients were screened, 68 apheresed, and 59 treated (Figure 2). Six patients discontinued prior to relma‐cel infusion due to disease progression (one death and five withdrawal) and three patients were at dose 25 × 10^6^ or 50 × 10^6^ CAR+ T‐cells. Cell product was manufactured for all apheresed patients, median production time was 19 days (range, 16–37). Treated patients had a median age of 56.0 years (range, 18–75); included several subtypes including 41 (69.5%) with Nonspecific diffuse large B‐cell lymphoma (DLBCL‐NOS), and six (10.2%) had previously undergone ASCT (Table 1). Sum of perpendicular diameters (SPD) ≥5000 mm^2^ was present in 14 (23.7%) of 59 patients; 26 (44.2%) in 59 patients had a bridging chemotherapy and 23 (47.9%) in 48 patients had a high‐risk International Prognostic Index (IPI) score ≥3 (Table 1). The median doses infused in low‐dose group and high‐dose group were 99.7 × 10^6^ (range, 80.1–101.3) and 150.0 × 10^6^ (range, 120.0–156.4) CAR+ T‐cells, respectively. No meaningful differences in baseline characteristics were apparent between dose groups (Table 1).

**TABLE 1:** Baseline characteristic.

Characteristics	100 × 10^6^ *N* = 27, *n* (%)		150 × 10^6^ *N* = 32, *n* (%)			Total *N* = 59, *n* (%)
Age (years)						
Median (range)	56.0 (19–71)		57.0 (18–75)			56.0 (18–75)
≥65 (years)	6 (22.2)		9 (28.1)			15 (25.4)
Sex						
Male	16 (59.3)		17 (53.1)			33 (55.9)
Female	11 (40.7)		15 (46.9)			26 (44.1)
Pathologic diagnosis						
DLBCL‐NOS	19 (70.4		22 (68.8			41 (69.5)
DLBCL with tFL	5 (18.5)		4 (12.5)			9 (15.3)
Primary mediastinal large B‐cell lymphoma	2 (7.4)		2 (6.3)			4 (6.8)
Grade 3B FL	1 (3.7)		1 (3.1)			2 (3.4)
DHL/THL	0		3 (9.4)			3 (5.1)
Tumor burden (SPD)						
<5000 mm^2^	20 (74.1)		25 (78.1)			45 (76.3)
≥5000 mm^2^	7 (25.9)		7 (21.9)			14 (23.7)
Immunohistochemistry type						
Double expression lymphoma (BCL−2 and MYC)	7 (25.9)		5 (15.6)			12 (20.3)
Non‐double expression lymphoma	15 (55.6)		19 (59.4)			34 (57.6)
IPI score^a^						
*n*	23		25			48
0–1	5 (21.7)		6 (24.0)			11 (22.9)
2	7 (30.4)		7 (28.0)			14 (29.2)
3	9 (39.1)		7 (28.0)			16 (33.3)
4–5	2 (8.7)		5 (20.0)			7 (14.6)
Bridging chemotherapy						
Yes	12 (44.4)		14 (43.8)			26 (44.2)
Previous immunogenic response						
Yes		0	0		0	
Relapse						
Yes		15 (55.6)		15 (46.9)		30 (50.8)
Refractory to the last therapy						
Yes		20 (74.1)		28 (87.5)	48 (81.4)	
Performance status (ECOG)						
0		11 (40.7)		13 (40.6)	24 (40.7)	
1–2^b^		16 (59.3)		19 (59.4)	35 (59.3)	
Line of prior therapy						
Median (range)		2 (2–7)		2 (2–7)	2 (2–7)	
2		15 (55.6)		17 (53.1)	32 (54.2)	
3–4		11 (40.7)		12 (37.5)	23 (39.0)	
≥5		1 (3.7)		3 (9.4)	4 (6.8)	
Autologous stem‐cell transplantation						
Yes		3 (11.1)		3 (9.4)	6 (10.2)	

Abbreviations: DHL/THL, high‐grade B‐cell lymphoma with MYC and BCL2 and/or BCL6 rearrangements; DLBCL‐NOS, diffuse large B‐cell lymphoma (NOS); IPI, international prognostic index; PMBCL, primary mediastinal large B‐cell lymphoma; tFL, non‐Hodgkin's lymphoma transformed recurrent.

^a^ 48 patients had the evaluation of IPI score.

^b^ One baseline ECOG score was 2.

### 3.2 Efficacy

**FIGURE 3:**
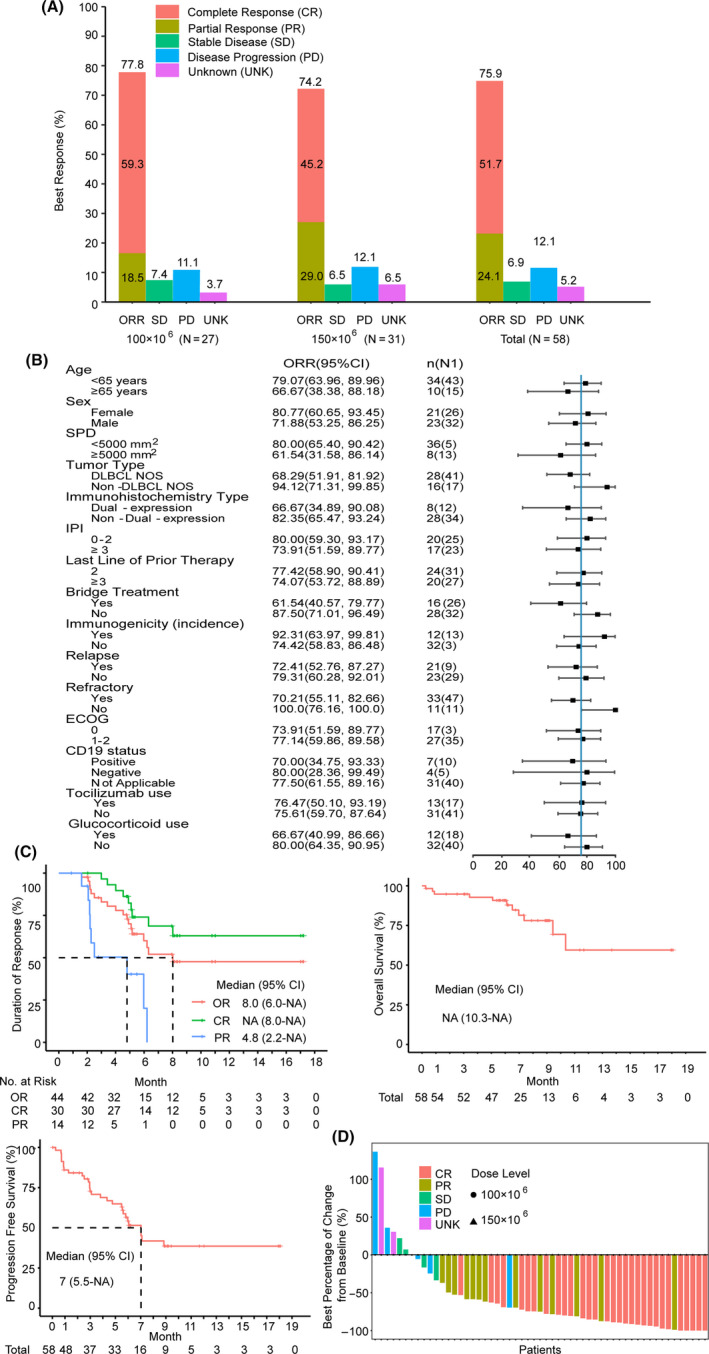
Efficacy assessed by investigators. A, BOR evaluation among the 58 patients who received relma‐cel infusion, an anti‐CD19 chimeric antigen receptor T‐cell therapy (ORR defined as CR plus PR). B, Forest plot showing the result of ORRs. CI=confidence interval. C, Kaplan–Meier curve of DOR among 44 patients, PFS and OS in the 58 patients. DOR, Time from the date of first documented disease response (CR or PR) to the date of first documented PD or death due to LBCL. PFS, Time from the date of relma‐cel infusion to the date of PD or death from 58 patients. OS, time from the date of relma‐cel infusion to the date of death. D, The change of tumor volume from the baseline according to SPD in 55 evaluable cases. The other four cases were excluded because they had clinical disease progression prior to the 1 month restaging assessments. The vertical axis indicates best percent changes from the baseline after relma‐cel infusion for 6 month. Patients are indicated on the horizontal axis. Negative values in the vertical axis indicate tumor volume decrease from the baseline. NA, Not Available.

Fifty‐eight patients were evaluable for efficacy outcomes, and the primary endpoint of 3 month ORR was 60.3% (95% CI, 46.6–73.0). The null hypothesis of a 3 month ORR ≤20% was rejected (*p* < 0.001). The excluded patient received product that did not meet potency threshold criterion, but achieved CR at D29 that continued for >1 year. As of data cutoff date, BOR rate was 75.9% (95% CI, 62.8–86.1) with a 51.7% CR rate (95% CI, 38.2–65.1; Figure 3A; Table S4). A sensitivity analysis was conducted using response restaging adjudicated by a prospectively established independent review committee (IRC) that yielded point estimates concordant with investigator assessment for both the primary endpoint and BOR rates (Table S3). High rates of response were observed across all key covariates, including age, SPD, IPI score at enrollment, ATA positive or negative post infusion, cell‐of‐origin subtype, Eastern Cooperative Oncology Group (ECOG), and use of tocilizumab or glucocorticoids (Figure 3B).

With median follow‐up of 8.9 months, median OS was not reached (95% CI, 10.3 to NA). Median PFS and DOR were 7.0 months (95% CI, 5.5 to NA) and 8.0 months (95% CI, 6.0 to NA), respectively, however, more than half of patients were censored prior to the median OS follow‐up (Figure 3C). In efficacy analysis set (58 patients), the median time to OR and CR evaluated by investigator were 0.92 and 0.95 month, respectively (Tables S6 and S7). Of 55 patients with baseline and at least one posttreatment imaging evaluation, the median best percentage change of SPD with treatment was −74.7% (range, −100–136.4%), with 42 (76.4%) patients having >50% decrease from baseline (Figure 3D).

### 3.3 Safety

Any grade adverse events (AEs) occurring in ≥10% of the 59 patients treated are shown in Table 2. Twenty‐eight patients (47.5%) experienced CRS of any grade. Grade 3 and 4 CRS was observed in two patients (3.4%) and one patient (1.7%), respectively (Tables 2 and 3). The median onset of CRS was at 4.5 days (range, 1–10) after infusion with a median duration of 7.0 days (range, 1–118; Table S5). Neurologic events occurred in 12 patients, with only 3 (5.1%) having severe grade events (all Grade 3; Tables 2 and 3). Median onset of NT was at 8.5 days after infusion, with a median duration of 12.5 days (range, 1–49; Table S5). CRS and NT were generally manageable and all cases resolved except one patient who died at day 8 due to sepsis with ongoing grade 4 CRS, but not related to CAR‐T cell therapy as evaluated by investigator.

**TABLE 2:** Treatment emergent adverse events.

	Any grade	Grade 3	Grade 4
Adverse event occurring in >10% patients			
Leukopenia	40 (67.80)	15 (25.42)	9 (15.25)
Neutropenia	38 (64.41)	14 (23.73)	13 (22.03)
Pyrexia	36 (61.02)	0	0
Lymphopenia	34 (57.63)	4 (6.78)	30 (50.85)
Blood immunoglobulin G decreased	28 (47.46)	0	0
Cytokine release syndrome	28 (47.46)	2 (3.39)	1 (1.69)
Blood immunoglobulin A decreased	26 (44.07)	0	0
Anemia	25 (42.37)	11 (18.64)	2 (3.39)
Thrombocytopenia	25 (42.37)	6 (10.17)	3 (5.08)
Blood immunoglobulin M decreased	22 (37.29)	0	0
Asthenia	11 (18.64)	0	0
Aspartate aminotransferase increased	8 (13.56)	2 (3.39)	0
Hypokalemia	8 (13.56)	0	0
Serum ferritin increased	8 (13.56)	1 (1.69)	0
Constipation	7 (11.86)	0	0
Tremor	7 (11.86)	2 (3.39)	0
Alanine aminotransferase increased	6 (10.17)	0	0
C‐reactive protein increased	6 (10.17)	0	0
Cough	6 (10.17)	0	0
Decreased appetite	6 (10.17)	0	0
Headache	6 (10.17)	0	0
Hypotension	6 (10.17)	2 (3.39)	0
Nausea	6 (10.17)	0	0
Cytokine release syndrome			
Pyrexia	23 (38.98)	0	0
Asthenia	5 (8.47)	0	0
Hypotension	4 (6.78)	2 (3.39)	0
C‐reactive protein increased	3 (5.08)	0	0
Decreased appetite	2 (3.39)	0	0
Dizziness	2 (3.39)	0	0
Headache	2 (3.39)	0	0
Nausea	2 (3.39)	0	0
Anoxia	1 (1.69)	0	1 (1.69)
Feeling cold	1 (1.69)	0	0
Malaise	1 (1.69)	0	0
Myalgia	1 (1.69)	0	0
Serum ferritin increased	1 (1.69)	1 (1.69)	0
Sinus tachycardia	1 (1.69)	0	0
Suffocation feeling	1 (1.69)	0	0
Temperature intolerance	1 (1.69)	0	0
Neurologic events			
Tremor	7 (11.86)	2 (3.39)	0
Neurotoxicity	3 (5.08)	0	0
Aphasia	2 (3.39)	1 (1.69)	0
Memory impairment	2 (3.39)	1 (1.69)	0
Cognitive disorder	1 (1.69)	0	0
Depressed level of consciousness	1 (1.69)	1 (1.69)	0
Disorientation	1 (1.69)	1 (1.69)	0
Dyscalculia	1 (1.69)	1 (1.69)	0
Headache	1 (1.69)	0	0
Hypersomnia	1 (1.69)	0	0
Hypoesthesia	1 (1.69)	0	0
Migraine	1 (1.69)	0	0
Slow response to stimuli	1 (1.69)	0	0
Somnolence	1 (1.69)	1 (1.69)	0

**TABLE 3:** The AEs of special interest occurred in the study, the usage of tocilizumab or glucocorticoids.

	100 × 10^6^ *N* = 27	150 × 10^6^ *N* = 32	Total *N* = 59
SAE, *n* (%)	5 (18.5)	9 (28.1)	14 (23.7)
CRS, *n* (%)			
Any	13 (48.1)	15 (46.9)	28 (47.5)
3	0	2 (6.3)	2 (3.4)
4	0	1 (3.1)	1 (1.7)
Neurotoxicity, *n* (%)			
Any	3 (11.1)	9 (28.1)	12 (20.3)
3	0	3 (9.4)	3 (5.1)
4	0	0	0
Infection			
Any	5 (18.5)	5 (15.6)	10 (17.0)
3	1 (3.7)	1 (3.1)	2 (3.4)
4	0	1 (3.1)	1 (1.7)
Febrile neutropenia, *n* (%)			
Any	1 (3.7)	2 (6.3)	3 (5.1)
3	1 (3.7)	2 (6.3)	3 (5.1)
4	0	0	0
Tumor lysis syndrome, *n* (%)			
Any	0	0	0
Death, *n* (%)^a^			
Total	3 (11.1)	8 (25.0)	11 (18.6)
Disease progression	3 (11.1)	7 (21.8)	10 (16.9)
Grade 5 toxicity	0	1 (3.1)	1 (1.7)
Tocilizumab, *n* (%)			
CRS	6 (22.2)	10 (31.3)	16 (27.1)
Neurotoxicity	0	1 (3.1)	1 (1.7)
Glucocorticoids, *n* (%)			
CRS	2 (7.4)	4 (12.5)	6 (10.1)
Neurotoxicity	0	3 (9.4)	3 (5.1)

Abbreviation: AE, adverse event; CRS, cytokine release syndrome; NT, neurotoxicity, SAE, serve adverse event.

^a^ None of the deaths were reported as related to relma‐cel.

Sixteen (27%) patients received tocilizumab and six (10%) corticosteroids for CRS, and one (1.7%) patient received tocilizumab and three (5.1%) corticosteroids for NT in accordance with the protocol‐defined toxicity management algorithm^19^ (Table 3).

### 3.4 Cellular kinetics

**FIGURE 4:**
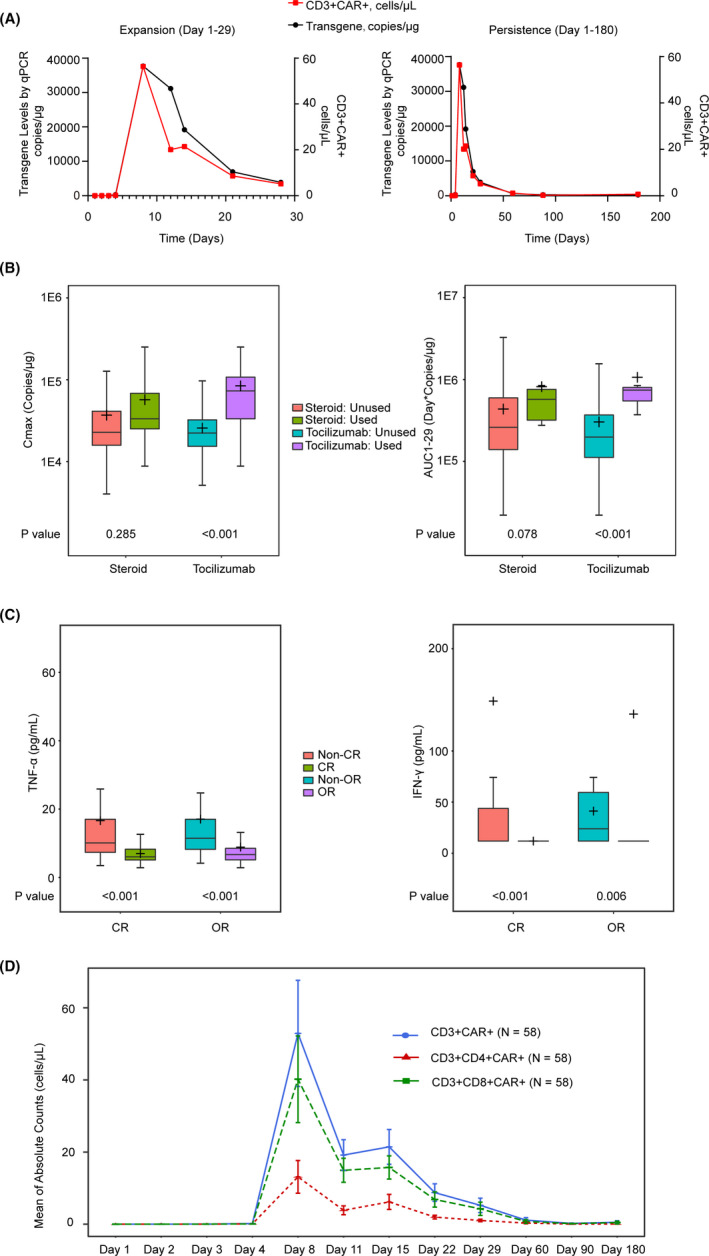
Cell Kinetic. A, CAR‐T expansion and persistence in peripheral blood were assessed via qPCR and flow cytometry on days 1, 2, 3, 4, 8, 11, 15, 22, and 29, monthly up to 3 months, every 3 months up to 1 year, then, every 6 months up to 2 years post infusion. The mean values were presented in the plots. B, The analysis of cellular kinetics exposure parameters (Cmax, AUC_1‐29_) for tocilizumab or steroid use. (N1: the number of subjects within the specific subgroup; *n*: the number of response subjects within the specific subgroup). C, The comparison of peak cytokines (IFN‐γ and TNF‐α) in patients with different therapeutic outcomes (OR/non‐OR and CR/non‐CR). D, The mean of absolute counts of CD3+CAR+, CD3+CD4+CAR+, and CD3+CD8+CAR+ over time from PB in total patients. IFN‐γ, Interferon gamma; TNF‐α, tumor necrosis factor alpha.

For 58 efficacy‐evaluable patients, median Cmax was 25333.5 copies/µg DNA by qPCR and 23.7 CD3+CAR+ cells/μL by flow cytometry with a median Tmax of 8.5 days and a median AUC_1‐29_ of 249744.8 day*copies/µg (Figure 4A; Tables S1 and S2). Correlations between PK parameters (Cmax, Tmax, and AUC) and selected clinical covariates (age, sex, ATA positive or negative post infusion, and corticosteroid or tocilizumab use) demonstrated no potential association except that higher Cmax and AUC were observed in those with tocilizumab use (Figure 4B; Figure S2). This is thought to be because patients with greater Cmax and AUC are more likely to develop sever CRS/NT and are therefore more likely to require tocilizumab therapy. Unlike a traditional drug, no relationship was detected between CAR‐T cell kinetics parameters and response (Figure S5). Although the relationship is generally expected for most drugs, the lack of a relationship here may be a result of the capacity of CAR‐T to proliferate in vivo and heterogeneity across a patient's product, native immune system, and tumor burden. Therefore, more studies are needed to confirm the hypothesis.

**FIGURE 5:**
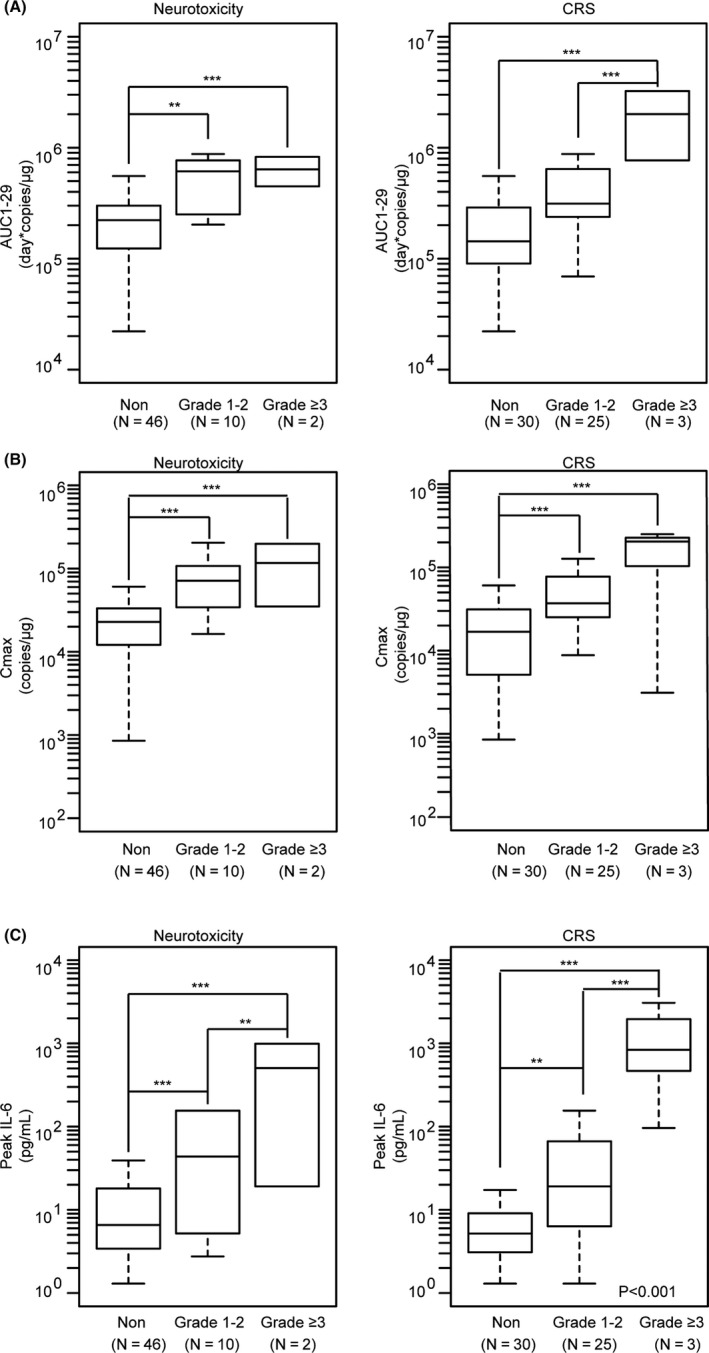
Analysis of serum biomarkers (CAR‐T expansion and Cytokines) associated with CRS/NT classified as none, grade 1–2 and grade ≥3 (**p < 0.01, ***p < 0.001). A, The comparison of AUC_1‐29_ and CRS/NT. B, The comparison of Cmax and CRS/NT. C, comparison of peak measured serum IL‐6 levels and CRS/NT. CRS, cytokine release syndrome; NT, neurotoxicity, IL‐6, Interleukin‐6. *p*‐values were calculated by means of the Wilcoxon rank‐sum test.

CD8+ and CD4+ CAR+ T‐cells showed similar PK profiles over time post infusion (Figure 4D) with both T‐cell subsets peaking around day 8 and the mean values of CD8+ T‐cells in the blood exceeding CD4+ T‐cells in the blood at each time point throughout the time course. Mean peak concentrations by flow for low‐ and high‐dose groups were dose proportional for CD4+ T‐cells, but were more than dose proportional for CD8+ T‐cells (Figure S1). No difference in exposure was observed between two dose groups. Higher Cmax for CAR+ T‐cells were observed in patients with any grade neurologic events and any grade CRS events and AUC_1‐29_ values for sever CRS and any grade NT (Figure 5A,B).

### 3.5 Serum biomarkers

Patients who achieved any response had lower baseline levels of serum TNF‐α and responding patients also had lower peak concentrations for TNF‐α and IFN‐γ (*p* < 0.01; Figure 4C). No association was observed between baseline levels, peak concentrations or the magnitude of change for other cytokine levels tested and disease response (Figure S3). Most peak serum cytokines (TNF‐α, MCP‐1, IL‐15, IL‐2, IFN‐γ, CRP, and ferritin) showed higher in those with serve CRS events, however, TGF‐β1 was lower in serve CRS. IL‐8 had no relationship in those with any grade CRS/NT (Figure S4). Peak serum IL‐6 levels were higher in those with any grade neurologic events and any grade CRS events, while IL‐15 was higher in serve CRS/NT (Figure 5C).

### 3.6 Anti‐treatment antibodies (ATA)

No pre‐infusion serum ATA was detected in any patient; post infusion, elevated serum ATA was observed in 10 (16.9%) patients at 6 months, among them five patients had complete remission (CR), three partial remission (PR), and two progressive disease (PD) at 6 months (data not shown).

## 4 DISCUSSION

This is the first prospective, single‐arm, multicenter, pivotal study of a CD19‐specific CAR‐T conducted under Chinese IND to support an NMPA‐accepted BLA submission in patients with r/r LBCL. In this study, relma‐cel demonstrated nearly 76% ORR and nearly 52% CRR in a cohort of r/r LBCL patients with many poor risk features, including 23.7% with high volume disease (SPD ≥5000 mm^2^), 44.2% requiring bridging chemotherapy for disease control, and 39.0% with IPI score ≥3. Many of these responses were durable given the current study follow‐up, with median DOR of 8 months with a plateau at approximately 48%. Toxicities commonly associated with anti‐CD19 CAR‐T were relatively low with any grade and severe CRS in 47.5% and 5.1%, respectively, and any grade and severe neurotoxicity in 20.3% and 5.1%, respectively. These low rates were obtained by using a protocol‐defined toxicity management algorithm, did not result from significant tocilizumab or corticosteroid use, which was required in only 28.8% and 15.2% of treated patients, respectively. Pharmacokinetic parameters showed similar expansion and contraction kinetics over time as has been reported by several groups.^20^, ^21^, ^22^, ^23^, ^24^

This trial is the first to randomize patients between two CAR‐T dosing groups, low dose (100 × 10^6^) and high dose (150 × 10^6^). While no overt improvement in response rates was observed, all severe CRS and NT events occurred with the higher dose. This suggests that higher cell dose could lead to higher severe toxicity rates, but not translate into higher response rates. Of note, CD8+ T‐cell subset expansion was also proportionately greater than CD4+ T‐cell subset expansion in the higher dose group, potentially suggesting an association with toxicity, as others have reported.^25^

The results of this trial compare with other multicenter registrational trial results in similar r/r LBCL populations. For example, the JULIET study,^8^ using tisagenlecleucel, demonstrated an ORR of 50%, CRR of 32%, and median DOR not reach with 9.4 months median follow‐up, but had severe CRS (Penn grading system) rate of 23% and severe neurologic event rate of 18%. Similarly, the ZUMA‐1 study^7^ evaluating axicabtagene ciloleucel, reported an ORR of 72%, CRR of 51%, and median DOR of 9.2 months, but with severe CRS (Lee grading system) and severe NT rates of 13% and 31%, respectively. Finally, the TRANSCEND trial, using lisocabtagene maraleucel, which shares the same CAR construct as relma‐cel, reported ORR of 73%, CRR of 53%, and median DOR of 13.3 months, with severe CRS and NT rates of 2% and 10%, respectively.^26^ Recently, The Center for International Blood and Marrow Transplant Research (CIBMTR) provided data on 410 patients receiving commercial tisagenlecleucel in the real‐world setting and an early analysis of safety and efficacy outcomes and reported that grade ≥3 CRS and NT were happened in 11.6% and 7.5% of all patients, respectively.^27^ In the RELIANCE trial, grade 3 or 4 adverse events of special interest included cytokine release syndrome (5.1% of the patients), infections (5.1%), neurologic events (5.1%), and febrile neutropenia (5.1%). Therefore, the efficacy of relma‐cel is essentially similar to other CAR‐T products and the special interested toxicities of relma‐cel are similar to 4‐1BB CAR‐T products (like tisagenlecleucel) but favorably to CD28 CAR‐T products such as axicabtagene ciloleucel.

Relative to other reported registrational CAR‐T studies in LBCL, anti‐cytokine therapy and corticosteroid use were similarly low and did not impact cell expansion or efficacy outcomes. Tocilizumab and corticosteroid use was 28.8% and 15.2%, respectively, for relma‐cel compared to these other CAR‐T trials reporting 19%–45% and 15%–27%, respectively. Use of tocilizumab and glucocorticoids did not significantly impact CAR‐T expansion in the peripheral blood of patients receiving these agents. Similarly, the use of tocilizumab or glucocorticoid also did not significantly reduce response rates which is the same as in previously reports.^28^, ^29^, ^30^

Anti‐CD19 CAR‐T therapy has been associated with significant serum levels of cytokines and biomarkers as a result of their expansion post infusion. Higher IL‐6 levels were observed in those with any grade CRS or NT as others have reported, while IL‐15 was higher in serve CRS/NT. Most peak serum cytokines (MCP‐1, TNF‐α, IL‐15, IL‐2, IFN‐γ, CRP, and ferritin) showed higher in those with serve CRS events except for IL‐8, however, TGF‐β1 was lower in serve CRS.

This is first demonstration of licensure‐quality CAR‐T manufacturing and multicenter clinical trial data with a CAR‐T development program completely originating in China. The durable response rates and safety profile observed with relma‐cel can offer a new option for Chinese adult patients with r/r LBCL. These study data have been submitted in a BLA and accepted by NMPA for review advancing both CAR‐T development in China and potentially access for LBCL patients to these novel therapies.

## CONFLICT OF INTEREST

No conflict of interest disclosures from authors.

## AUTHOR CONTRIBUTIONS

Jun Zhu: Project administration, patient enrollment, data acquisition, and article review. Zhitao Ying: Patient enrollment, data acquisition, patient evaluation, and article preparation. Yuqin Song: Data acquisition, statistical analysis, blood sample collection, and patient evaluation. Haiyan Yang, Ye Guo, Wenyu Li, Dehui Zou, Daobin Zhou, Zhao Wang, Mingzhi Zhang, and Jianqiu Wu: Patient enrollment and data acquisition. Hui Liu: Patient enrollment. Pian Zhang, Su Yang, Zisong Zhou, and Hongxia Zheng: Project administration and supervision.

## FUNDING INFORMATION

All funding provided by JW Therapeutics (Shanghai) Co. Ltd.

## Supporting information

Supplementary Material

## Data Availability

The data that support the findings of this study are available from the corresponding author upon reasonable request.
