# Supplementary material for: Relmacabtagene autoleucel (relma‐cel) CD19 CAR‐T therapy for adults with heavily pretreated relapsed/refractory large B‐cell lymphoma in China
Source: Cancer Med. 2020 Dec 31;10(3):999–1011. doi: 10.1002/cam4.3686 (PMC7897944; doi:10.1002/cam4.3686)
Supplement: Supplementary file 1 — Supplementary Material [file CAM4-10-999-s001.docx]

**Supporting Information**

**Table S1.** Pharmacokinetic parameters of relma-cel by flow cytometry

|  | 100×10^6^  N=27 | 150×10^6^  N=31 | Total  N=58 |  |
| --- | --- | --- | --- | --- |
| **Cmax (cells/μL)** | | | |  |
| n | 27 | 31 | 58 |  |
| Median  Min, Max | 15.8  2.4, 288.52 | 31.5  (0.6, 582.0) | 23.7  (0.6, 582.0) |  |
| **Tmax (day)** | | | |  |
| n | 27 | 31 | 58 |  |
| Median  Min, Max | 10.0  8, 40 | 9.0  4, 20 | 9.0  4, 40 |  |
| **AUC_1-29_ (day*cells/μL)** | | | |  |
| n | 23 | 28 | 51 |  |
| Median  Min, Max | 138.3  21.6, 2338.5 | 319.7  58.8, 2936.4 | 271.5  21.6, 2936.4 |  |
| Abbreviation: AUC, area under the curve; Cmax, maximum concentration; Min, minimum; Max, maximum; qPCR, quantitative polymerase chain reaction; Tmax, Time to maximum concentration. | | | | |

**Table S2.** Pharmacokinetic Parameters of relma-cel by qPCR

|  | 100×10^6^  N=27 | 150×10^6^  N=31 | Total N=58 |
| --- | --- | --- | --- |
| **Cmax (copies/μg)** | | | |
| n | 27 | 31 | 58 |
| Median  Min, Max | 22983.0  1598, 127171 | 25451.0  854, 250768 | 25333.5  854, 250768 |
| **Tmax (day)** | | | |
| n | 27 | 31 | 58 |
| Median  Min, Max | 9.0  7, 27 | 8.0  4, 22 | 8.5  4, 27 |
| **AUC_1-29_ (day*copies/μg)** | | | |
| n | 24 | 28 | 52 |
| Median  Min, Max | 240572.2  22089.3, 2218744.8 | 265602.67  22678.0, 3241025.5 | 249744.80  22089.3, 3241025.5 |
| Abbreviation: AUC, area under the curve; Cmax, maximum concentration; Min, minimum; Max, maximum; qPCR, quantitative polymerase chain reaction; Tmax, Time to maximum concentration. | | | |

**Table S3.** Clinical response evaluated by IRC

|  | 100×10^6^  N=27 | 150×10^6^  N=31 | Total  N=58 |
| --- | --- | --- | --- |
| **3 Month** | | | |
| ORR, n (%) | 19 (70.4) | 16 (51.6) | 35 (60.3) |
| ORR, (95% CI) | (49.8, 86.3) | (33.1, 69.9) | (46.6, 73.0) |
| CR, n (%) | 18 (66.7) | 12 (38.7) | 30 (51.7) |
| CR, (95% CI) | (46.0, 83.5) | (21.9, 57.8) | (38.2, 65.1) |
| **Best Objective Response** | | | |
| ORR, n (%) | 22 (81.5) | 22 (71.0) | 44 (75.9) |
| ORR, (95% CI) | (61.9, 93.7) | (52.0, 85.8) | (62.8, 86.1) |
| CR, n (%) | 19 (70.4) | 15 (48.4) | 34 (58.6) |
| CR, (95% CI) | (49.8, 86.3) | (30.2, 66.9) | (44.9, 71.4) |
| Abbreviation: CRR, complete remission rate; CI, confidence interval; IRC, independent review committee; ORR, objective response rate. | | | |

**Table S4.** Clinical response evaluated by investigators

|  | 100×10^6^  N=27 | 150×10^6^  N=31 | Total  N=58 |
| --- | --- | --- | --- |
| **3 Month** | | | |
| ORR, n (%) | 18 (66.7) | 17 (54.8) | 35 (60.3) |
| ORR, (95% CI) | (46.0, 83.5) | (36.0, 72.7) | (46.6, 73.0) |
| CR, n (%) | 16 (59.3) | 10 (32.3) | 26 (44.8) |
| CR, (95% CI) | (38.8, 77.6) | (16.68, 51.4) | (31.7, 58.5) |
| **Best Objective Response** | | | |
| ORR, n (%) | 21 (77.8) | 23 (74.2) | 44 (75.9) |
| ORR, (95% CI) | (57.7, 91.4) | (55.4, 88.1) | (62.8, 86.1) |
| CR, n (%) | 16 (59.3) | 14 (45.2) | 30 (51.7) |
| CR, (95% CI) | (38.8, 77.6) | (27.3, 64.0) | (38.2, 65.1) |
| Abbreviation: CRR, complete remission rate; CI, confidence interval; ORR, objective response rate. | | | |

**Table S5.** Time to CRS/NT occurred and CRS/NT duration

|  | 100×10^6^  N=27 | 150×10^6^  N=32 | Total  N=59 |
| --- | --- | --- | --- |
| **CRS** | | | |
| **Time to CRS Occurred (Days)** | | | |
| n | 13 | 15 | 28 |
| Median  Min, Max | 4.0  1, 7 | 5.0  1, 10 | 4.0  1, 10 |
| P25 | 4.0 | 2.0 | 2.5 |
| P75 | 10.0 | 11.0 | 10.5 |
| **Duration (Days)** | | | |
| n | 13 | 15 | 28 |
| Median  Min, Max | 6.0  1, 118 | 7.0  3, 19 | 7.0  1, 118 |
| P25 | 4.0 | 2.0 | 2.5 |
| P75 | 6.0 | 5.0 | 6.0 |
| **Neurotoxicity** | | | |
| **Time to NT Occurred (Days)** | | | |
| n | 3 | 9 | 12 |
| Median  Min, Max | 7.0  6, 7 | 9.0  2, 11 | 8.5  2, 11 |
| P25 | 7.0 | 10.0 | 7.0 |
| P75 | 7.0 | 11.0 | 11.0 |
| **Duration (Days)** | | | |
| n | 3 | 9 | 12 |
| Median  Min, Max | 14.0  5, 17 | 11.0  1, 49 | 12.5  1, 49 |
| P25 | 5.0 | 1.0 | 1.0 |
| P75 | 5.0 | 5.0 | 5.0 |
| Abbreviation: CRS, cytokine release syndrome; NT, neurotoxicity. | | | |

**Table S6:** Time to first objective response and complete response evaluated by investigator

|  | **100×10^6^ (N=27)** | **150×10^6^ (N=31)** | **Total (N=58)** |
| --- | --- | --- | --- |
| **Number of Objective Response, n (%)** | 21(77.8) | 24(77.4) | 45(77.6) |
| **Time to First Objective Response (Month)** |  |  |  |
| Mean (Std) | 0.879(0.0886) | 1.474(2.0298) | 1.196(1.4991) |
| Median (Min, Max) | 0.920(0.72,1.08) | 0.920(0.79,10.58) | 0.920(0.72,10.58) |
| Q1, Q3 | 0.820,0.920 | 0.820,1.005 | 0.820,0.950 |
| **Number of Complete Response, n (%)** | 16(59.3) | 14(45.2) | 30(51.7) |
| **Time to First Complete Response (Month)** |  |  |  |
| Mean (Std) | 1.578(0.9406) | 1.798(1.9340) | 1.681(1.4652) |
| Median (Min, Max) | 0.950(0.76,3.02) | 0.935(0.79,6.28) | 0.950(0.76,6.28) |
| Q1, Q3 | 0.885,2.695 | 0.850,1.050 | 0.850,2.600 |

**Table S7:** Time to first objective response and complete response evaluated by IRC

|  | **100×10^6^ (N=27)** | **150×10^6^ (N=31)** | **Total (N=58)** |
| --- | --- | --- | --- |
| **Number of Objective Response, n (%)** | 22(81.5) | 22(71.0) | 44(75.9) |
| **Time to First Objective Response (Month)** |  |  |  |
| Mean (Std) | 0.885(0.0878) | 0.985(0.4220) | 0.935(0.3055) |
| Median (Min, Max) | 0.920(0.72,1.08) | 0.905(0.79,2.83) | 0.920(0.72,2.83) |
| Q1, Q3 | 0.820,0.950 | 0.820,0.990 | 0.820,0.950 |
| **Number of Complete Response, n (%)** | 19(70.4) | 15(48.4) | 34(58.6) |
| **Time to First Complete Response (Month)** |  |  |  |
| Mean (Std) | 1.357(0.8541) | 1.469(1.3729) | 1.406(1.0958) |
| Median (Min, Max) | 0.920(0.76,3.02) | 0.850(0.79,5.59) | 0.920(0.76,5.59) |
| Q1, Q3 | 0.820,1.970 | 0.790,1.050 | 0.820,1.080 |

**Figure S1.** CD3+CD4+CAR+ and CD3+CD8+CAR+ T-cell counts in two dose groups


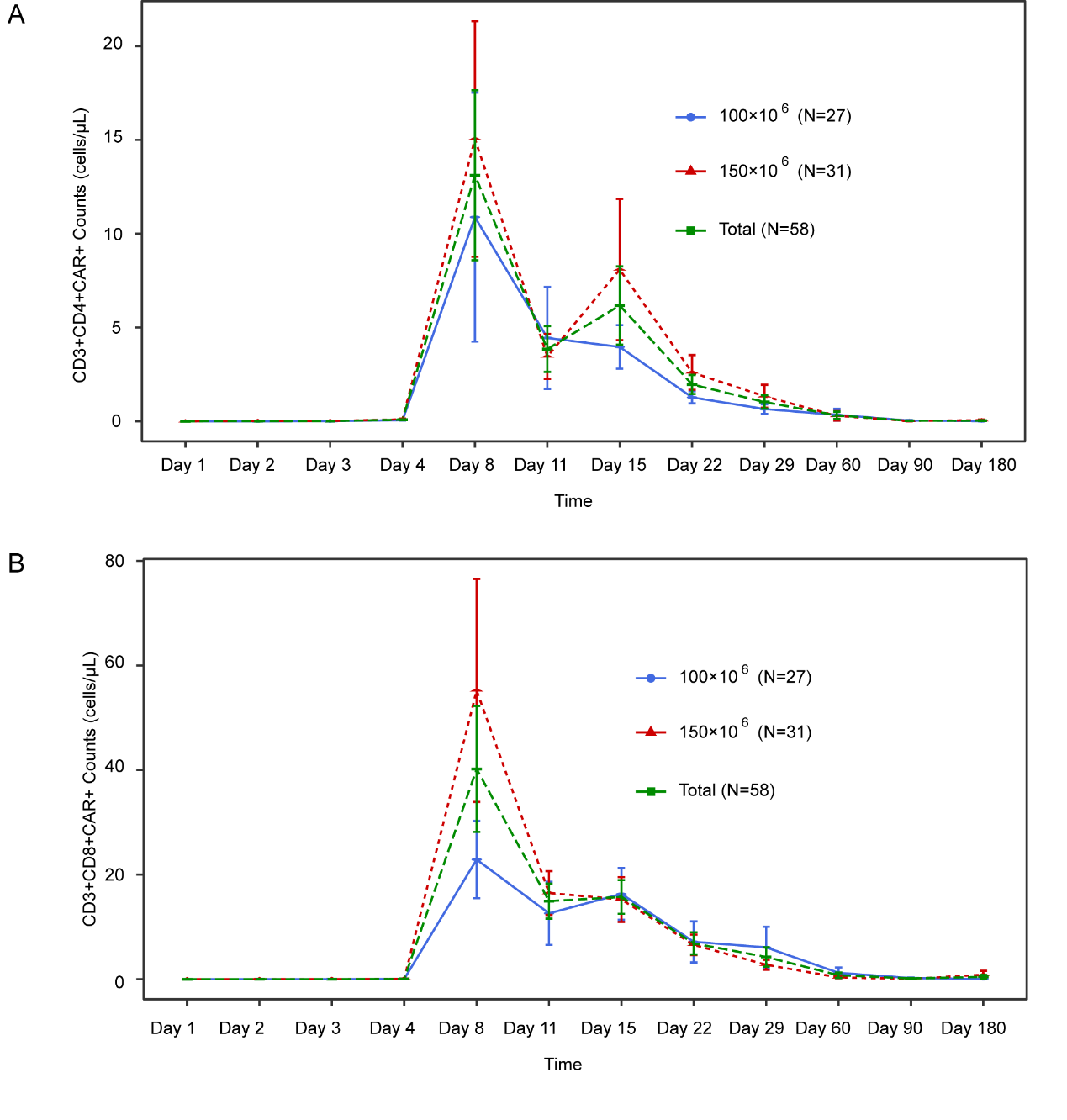


**Figure S2.** The analysis of cellular kinetics exposure parameters (Cmax, Tmax and AUC_1-29_,)


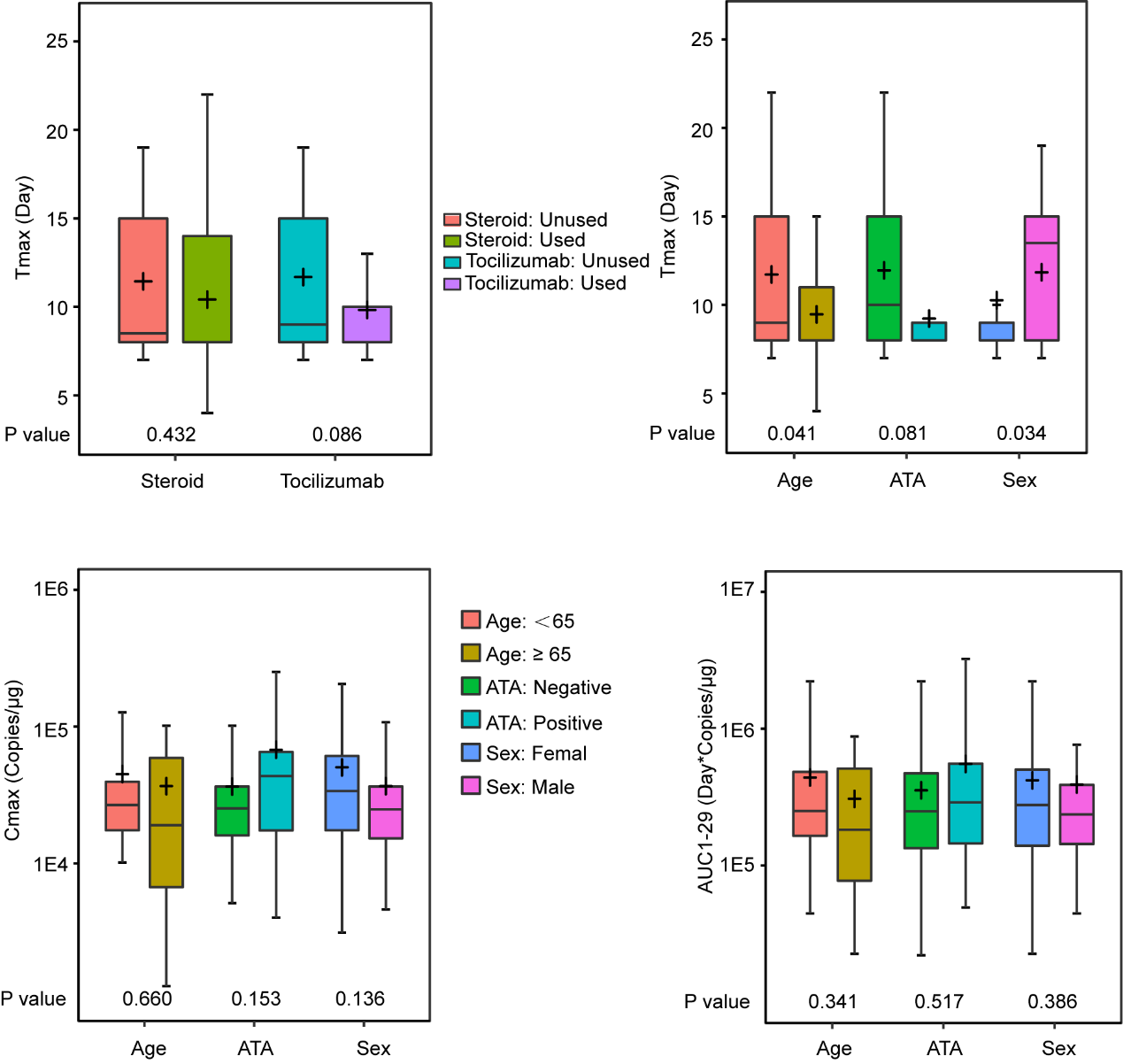


**Figure S3.** The comparison of peak biomarkers (IL-2, IL-6, IL-8, IL-15, MCP-1, TGF-β1, CRP and ferritin) in patients with different therapeutic outcomes (OR/non-OR and CR/non-CR)


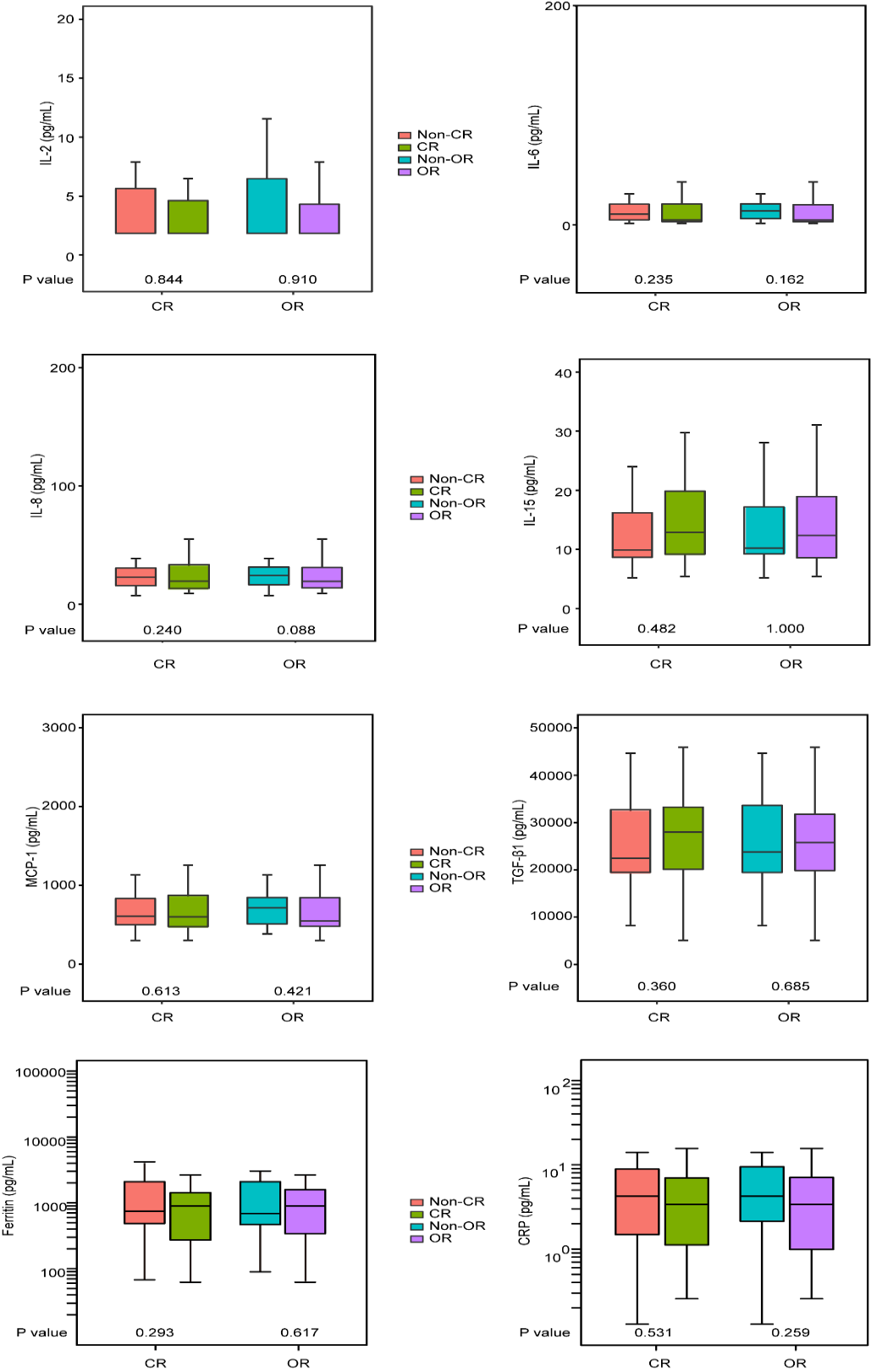


**Figure S4.** The comparison of peak serum biomarkers (TNF-α, TGF-β1, MCP-1, IL-2, IL-8, IL-15, IFN-γ, CRP and ferritin) with CRS/NT (** *P* <0.01, *** *P* <0.001)


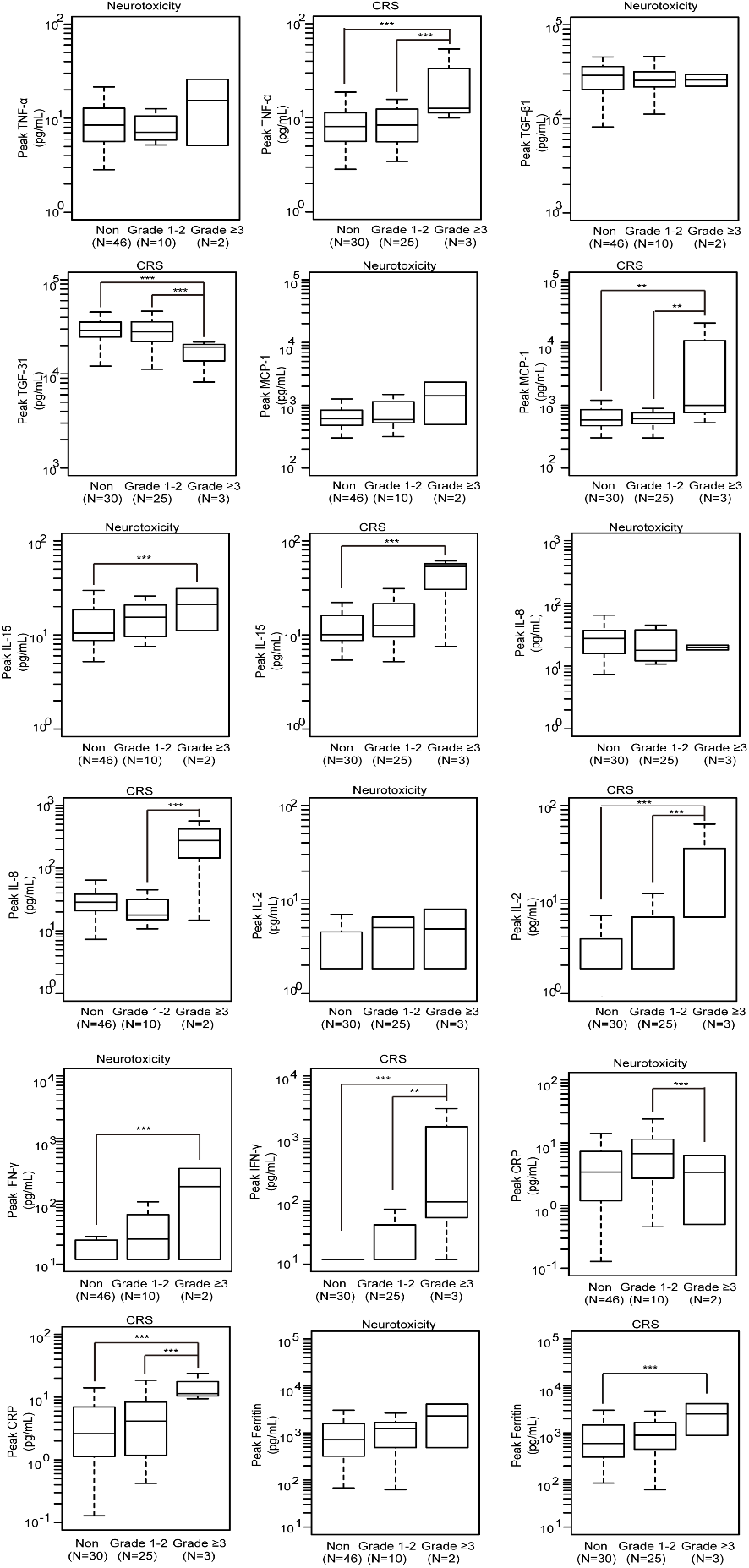


**Figure S5.** The correlations between relma-cel kinetics parameters (Cmax, Tmax and AUC_1-29_) and response

**
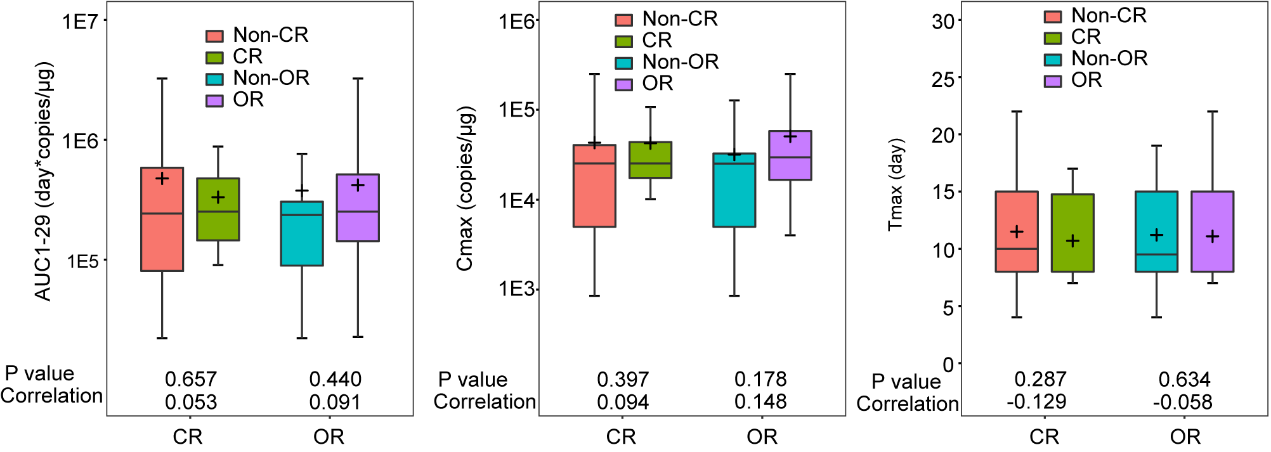
**

**Figure legends:**

**Figure S1.** CD3+CD4+CAR+ and CD3+CD8+CAR+ T-cell counts in two dose groups. A, CD3+CD4+CAR+ T-cell count change over time from peripheral blood in low dose group (blue), high dose group (red) and total patients (green). B, CD3+CD8+CAR+ T-cell count change over time from peripheral blood in low dose group (blue), high dose group (red) and total patients (green).

**Figure S2.** The relationship between PK exposure parameters (Cmax, Tmax and AUC_1-29_) and key factors (age, sex and ATA positive or negative post infusion). P values were calculated by means of the Wilcoxon rank-sum test. The horizontal line within each box represents the median, and the lower and upper borders of each box represent the 25th and the 75th percentiles, respectively.

Abbreviation: ATA, anti-therapeutic antibodies. PK, [pharmacokinetics](file:///C:\Users\nicky.ni\AppData\Local\youdao\dict\Application\7.2.0.0511\resultui\dict\?keyword=pharmacokinetics)

**Figure S3.** Serum biomarkers (IL-2, IL-6, IL-8, IL-15, MCP-1, TGF-β1, CRP and ferritin) that were associated with different therapeutic outcomes (OR/non-OR and CR/non-CR). The peak value is defined as the maximum level of the cytokine after baseline. *P* values were calculated by means of the Wilcoxon rank-sum test. The horizontal line within each box represents the median, and the lower and upper borders of each box represent the 25th and the 75th percentiles, respectively.

Abbreviation: IL-2, interleukin-2; IL-6, interleukin-6; IL-8, interleukin-8; IL-15, interleukin-15; TGF-β1, transforming growth factor-β1, TNF-α, tumor necrosis factor alpha, IFN-γ, interferon gamma; MCP-1, monocyte chemotactic protein 1 and CRP C-reactive protein.

**Figure S4.** Serum biomarkers (TNF-α, TGF-β1, MCP-1, IL-2, IL-8, IL-15, IFN-γ, CRP and ferritin) that were associated with neurologic events and the cytokine release syndrome. The peak value is defined as the maximum level of the cytokine after baseline. *P* values were calculated by means of the Wilcoxon rank-sum test. The horizontal line within each box represents the median, and the lower and upper borders of each box represent the 25th and the 75th percentiles, respectively. (** *P* <0.01, *** *P* <0.001).

**Figure S5.** The correlations between relma-cel kinetics parameters (Cmax, Tmax and AUC_1-29_) and response. *P* values were calculated by means of the Wilcoxon rank-sum test. The horizontal line within each box represents the median, and the lower and upper borders of each box represent the 25th and the 75th percentiles, respectively. (** *P* <0.01, *** *P* <0.001).

**List of participating sites**

1. Department of Lymphoma, Key Laboratory of Carcinogenesis and Translational Research (Ministry of Education/Beijing), Peking University Cancer Hospital & Institute, Beijing, China
2. Department of Lymphatic Medical, Cancer Hospital of the University of Chinese Academy of Sciences, Zhejiang, China
3. Department of Oncology, Shanghai East Hospital, Shanghai, China
4. Department of Lymphoma, Guangdong Academy of Medical Sciences, Guangdong Provincial People’s Hospital, Guangdong, China
5. Lymphoma Center, Institute of Hematology & Blood Diseases Hospital Chinese Academy of Medical Sciences & Peking Union Medical College Institute of Hematology and Blood Diseases Hospital (IH), Tianjin, China
6. Department of Hematology, Peking Union Medical College Hospital, Chinese Academy of Medical Sciences, Beijing, China
7. Department of Hematology, Beijing Friendship Hospital, Capital Medical University, Beijing, China
8. Department of Oncology, the First Affiliated Hospital of Zhengzhou University, Zhengzhou, China
9. Department of Medical Oncology, Jiangsu Institute of Cancer Research, Jiangsu Red Cross Cancer Center, Jiangsu Cancer Hospital, the Affiliated Hospital of Nanjing Medical University, Nanjing, China
10. Department of Hematology, Beijing Hospital, Beijing, China
